# VirtualCytometry: a webserver for evaluating immune cell differentiation using single-cell RNA sequencing data

**DOI:** 10.1093/bioinformatics/btz610

**Published:** 2019-08-02

**Authors:** Kyungsoo Kim, Sunmo Yang, Sang-Jun Ha, Insuk Lee

**Affiliations:** Department of Biotechnology, Yonsei University, Seoul 03722, Korea; Department of Biotechnology, Yonsei University, Seoul 03722, Korea; Department of Biochemistry, Yonsei University, Seoul 03722, Korea; Department of Biotechnology, Yonsei University, Seoul 03722, Korea; Department of Biomedical Systems Informatics, Yonsei University College of Medicine, Seoul 03722, Korea

## Abstract

**Motivation:**

The immune system has diverse types of cells that are differentiated or activated via various signaling pathways and transcriptional regulation upon challenging conditions. Immunophenotyping by flow and mass cytometry are the major approaches for identifying key signaling molecules and transcription factors directing the transition between the functional states of immune cells. However, few proteins can be evaluated by flow cytometry in a single experiment, preventing researchers from obtaining a comprehensive picture of the molecular programs involved in immune cell differentiation. Recent advances in single-cell RNA sequencing (scRNA-seq) have enabled unbiased genome-wide quantification of gene expression in individual cells on a large scale, providing a new and versatile analytical pipeline for studying immune cell differentiation.

**Results:**

We present VirtualCytometry, a web-based computational pipeline for evaluating immune cell differentiation by exploiting cell-to-cell variation in gene expression with scRNA-seq data. Differentiating cells often show a continuous spectrum of cellular states rather than distinct populations. VirtualCytometry enables the identification of cellular subsets for different functional states of differentiation based on the expression of marker genes. Case studies have highlighted the usefulness of this subset analysis strategy for discovering signaling molecules and transcription factors for human T-cell exhaustion, a state of T-cell dysfunction, in tumor and mouse dendritic cells activated by pathogens. With more than 226 scRNA-seq datasets precompiled from public repositories covering diverse mouse and human immune cell types in normal and disease tissues, VirtualCytometry is a useful resource for the molecular dissection of immune cell differentiation.

**Availability and implementation:**

www.grnpedia.org/cytometry

## 1 Introduction

The immune system is composed of diverse cell types that have different roles during infection, disease and development. Under challenged conditions, immune cells are activated and differentiated by remodeling of their epigenetic landscape enabling them to exert their functions. Key signaling molecules and transcription factors in immune cell differentiation have been investigated by immunophenotyping with flow cytometry (Adan *et al.*, 2017) and mass cytometry (Spitzer and Nolan, 2016), which can quantify cell-to-cell variations in gene expression at the protein level. Although these technologies have provided valuable insights, the limited number of proteins that can be probed in a single experiment prevents obtaining a more comprehensive view of the molecular networks involving in immune cell differentiation.

Cells are the functional units of multicellular organisms. However, gene expression analysis using next-generation sequencing technology is typically conducted using tissue samples in a process commonly referred to as bulk RNA-seq. Because tissues are generally composed of a wide variety of cell types in different developmental states, the observed transcriptome profile may be the average signals of a heterogeneous cell population or proxy of the dominant cell type. This problem of cellular heterogeneity in gene expression studies can be overcome by single-cell RNA sequencing (scRNA-seq), which empowers cell biology by enabling the identification of rare cell types, reconstruction of the dynamics of transcriptional states during cellular development, mapping of individual cells in tissue space and remodeling of regulatory networks underlying cellular phenotypes (Regev *et al.*, 2017; Tanay and Regev, 2017). In the past several years, the amount of scRNA-seq data obtained for human and mouse immune cells in public repositories has greatly expanded (Papalexi and Satija, 2018). Therefore, exploiting cell-to-cell variations in gene expression with scRNA-seq may enable analysis of immune cell differentiation in diverse contexts and tissues (Griffiths *et al.*, 2018).

**Fig. 1.**
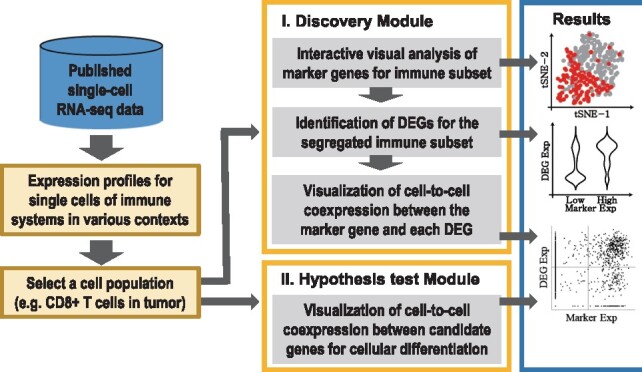
Overview of VirtualCytometry workflow

In this study, we developed VirtualCytometry (www.grnpedia.org/cytometry), a web-based computational pipeline for examining immune cell differentiation using scRNA-seq data (Fig. 1). Cells in developmental states belonging to distinct transcriptional populations can be evaluated to identify differentiation-associated genes based on gene expression between populations. This conventional approach is also employed by other web-based scRNA-seq data analysis tools (Abugessaisa *et al.*, 2018; Cao *et al.*, 2017). However, differentiating cells often show a continuous spectrum of cellular states rather than distinct populations. In that case, we need an alternative approach to identify genes involved in cellular differentiation. Distinct cell types and states can be often observed by using single marker genes. Thus, we hypothesized that comparative analysis of cellular subsets divided based on marker expression levels would be useful for identifying differentially expressed genes (DEGs) between subsets that potentially mediate their differentiation.

In particular, the data analysis pipeline of VirtualCytometry mimics the subset analysis strategy used to analyze data from flow or mass cytometry. It enables users to divide a given cell population into two subsets according to the user-selected marker expression level, and then identify DEGs between subsets based on non-parametric Wilcoxon test. DEGs showing a strong correlation with marker expression are likely strong candidate genes involved in the transition between the types or functional states of immune cells. Although correlation analysis with scRNA-seq data is affected by the highly noisy and zero-inflated nature of the data (i.e. a majority of the measured gene expressions have zero values), population-wise correlations may provide reasonably robust signals of functional coupling between two genes during cellular differentiation or activation.

VirtualCytometry was tested to identify signaling molecules and transcription factors involved in human CD8^+^ T-cell exhaustion, a state of T-cell dysfunction, in tumors and the mouse dendritic cell (DC) response after pathogen activation.

## 2 Materials and methods

### 2.1 Single-cell RNA sequencing datasets

**Fig. 2.**
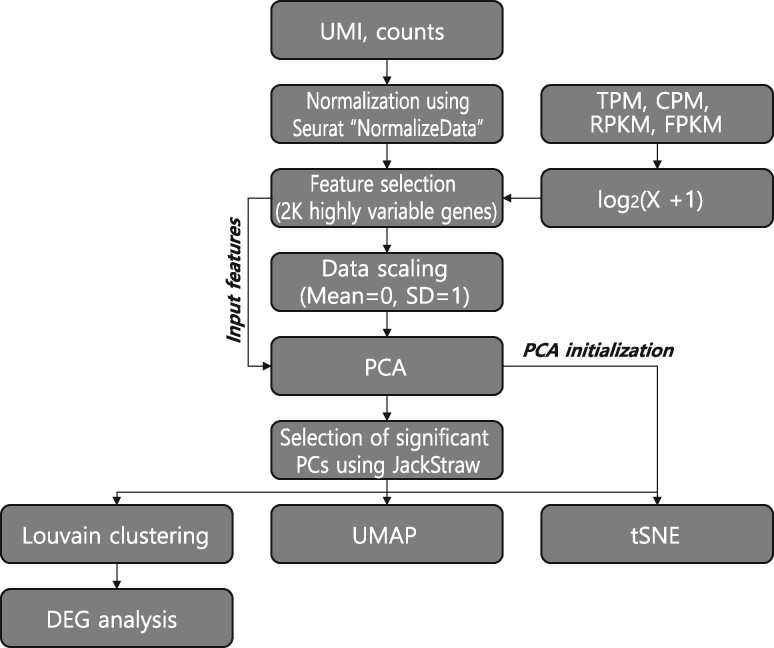
Overview of single-cell RNA sequencing data pre-processing, feature selection and visualization analysis for VirtualCytometry

The web-based analysis pipeline used scRNA-seq datasets of cells related to hematopoiesis and immunity in JingleBells (Ner-Gaon *et al.*, 2017) and Gene Expression Omnibus (GEO) (Barrett *et al.*, 2013). We searched the GEO database for the term ‘single cell’ and selected series of the type ‘Expression profiling by high throughput sequencing’. We excluded datasets that included cells without cell type labels because VirtualCytometry was designed to examine cellular differentiation within known distinct cell types. Single-cell RNA sequencing data obtained from public depositories were subjected to our pipeline for data pre-processing and feature selection and visualization as summarized in Figure 2.

Some datasets provide only raw sequence read data for each cell. Thus, we only used datasets that included quantitative values. Single-cell RNA-seq data deposited in public databases are available along with various quantitative values. For datasets with UMI (unique molecular identifier) or count values, we filtered out poor-quality cells and performed normalization using the function NormalizeData of the Seurat package (Butler *et al.*, 2018). For datasets with normalized count values such as CPM (counts per million), TPM (transcripts per million), RPKM (reads per kilobase per million) or FPKM (fragments per kilobase per million), the log-like transformation log_2_(*X* + 1) was applied, in which *X* is the normalized count values. Finally, we excluded datasets when the labeled cell-type had no more than 16 cells, which is too small to give an appropriate perplexity value for dimension reduction (see Section 2.2). We organized the precompiled datasets for each cell type along with associated context (normal tissue, disease tissue, pathogen stimulation, etc.) for mouse and human.

Owing to the highly zero-inflated and noisy nature of single-cell RNA-seq data, downstream analysis was improved by feature selection and data scaling. We selected only 2000 highly variable genes for downstream analysis using the FindVariableFeatures function of the Seurat package. We then converted the mean and standard deviation of normalized count values to ‘0’ and ‘1’ using the ScaleData function of the Seurat package.

Cell cycle phase is often considered a confounding factor in scRNA-seq data analysis (Buettner *et al.*, 2015). However, we did not explicitly remove cell-cycle effects during analysis because the cell-cycle phase can be associated with different cellular states. For example, many immune cell subtypes have different proliferation rates as important characteristics.

### 2.2 Data visualization

To explore subpopulations in the given dataset using gene expression kinetics, we employed a dimension reduction method, t-Distributed Stochastic Neighbor Embedding (tSNE) (van der Maaten and Hinton, 2008) and UMAP (Becht *et al.*, 2019). Coordinates of tSNE plot were calculated using the Rtsne package. To calculate UMAP coordinates, we used the RunUMAP function of the Seurat package with the same input dimensions as the tSNE analysis.

For tSNE, two important parameters were the number of input dimensions to be used and perplexity. It is highly recommended to use PCA to reduce the number of dimensions for tSNE, thereby suppressing some noise in the original data. Principal component analysis (PCA) was performed using 2000 genes with highly variable expression. To identify statistically significant principal components (PCs), we used the JackStraw test (Chung and Storey, 2015) available in the Seurat package. We selected all PCs starting from the first PC to the one before encountering a non-significant PC (*P* > 0.05). However, datasets could have either only a few significant PCs or more than a hundred. Downstream analysis of tSNE based on a small number of PCs is biased, and noise affects that based on too many PCs. Therefore, we limited the range of input dimensions for the tSNE analysis to between 10 and 50.

Perplexity governs how many nearest neighbors can be attracted to each data point, affecting the local and global structures of the tSNE output. A smaller perplexity shows more local structures whereas a larger perplexity shows more global data structures, and typical values are between 5 and 50 (van der Maaten and Hinton, 2008). For VirtualCytometry, we used 30 as the perplexity value. In addition, we used PCA initialization that uses the first two PCs to maintain the global structure of the tSNE output (Kobak and Berens, 2018). For datasets with (N-1)/3<30, where *N* = the number of cells, the tSNE analysis software cannot perform the computation. Thus, in that case, we rounded (N-1)/3 down to an integer and used it as the perplexity value.

VirtualCytometry was designed to perform analysis with a user-selected marker gene. However, if users do not have pre-selected marker genes, VirtualCytometry can suggest candidate markers via differential expression analysis for predefined clusters of cells. We defined clusters of cells using the Louvain clustering algorithm implemented as the FindNeighbors and FindClusters functions of the Seurat package with 10 different resolution parameters in the range spanning from 0.1 to 1.0 and the same number of PCs as the dimension reduction analysis. Next, we identified candidate marker genes for each cluster using the FindAllMarkers function of the Seurat package, followed by filtering genes that were expressed in fewer than 10% of cells, have a log fold-change value of 0.25 or higher, and an adjusted *P-*value <0.05.

### 2.3 Differential expression analysis

To evaluate whether each gene was differentially expressed between the two subsets determined by the marker gene expression level, we employed non-parametric Wilcoxon test. We implemented the webserver backend to calculate adjusted *P*-values using the p.adjust function in R package and output only genes with adjusted *P*-values <0.05 as DEGs. The resultant DEGs were sorted based on the largest mean fold-change.

### 2.4 List of transcription factors and surface proteins

We compiled 2765 human and 1726 mouse TFs from previous studies (Lambert *et al.*, 2018; Ravasi *et al.*, 2010). Human surface proteins were obtained from CellPhoneDB (Vento-Tormo *et al.*, 2018) with annotation of ‘Transmembrane’ or ‘Peripheral’ in their curated information. We compiled lists of mouse surface proteins from cellular component annotations in the UniProt and Gene Ontology databases. We selected genes annotated as ‘cell membrane’ and ‘cell surface’ in UniProt and those as ‘external side of plasma membrane’, ‘external side of cell outer membrane’ and ‘cell outer membrane’. For Gene Ontology annotations, we considered only those with experimental or literature evidence (GO evidence code of IDA, IPI, IMP, IGI, IEP, TAS and EXP). Finally, we obtained 3674 and 2608 surface proteins for human and mouse, respectively.

### 2.5 Discovery module

Novel genes involved in immune cell differentiation can be predicted with the ‘Discovery module’ of VirtualCytometry. This module divides a given cell population into two subsets based on marker gene expression levels and predicts DEGs between the subsets as candidates associated with differentiation process in the following four steps.

1. *Selection of a cell population for analysis*: Users must select a precompiled scRNA-seq dataset of cell populations. VirtualCytometry provides more than 226 scRNA-seq datasets for immune cells from human and mouse. Each dataset is annotated by tissue origin, cell type/subtype, disease and other descriptions. The webserver first visualizes the user-selected cell population in either a tSNE plot (van der Maaten and Hinton, 2008) or a UMAP plot (Becht *et al.*, 2019).
2. *Interactive visual analysis of marker genes for subset segregation*: Users can select a marker gene for the analysis either based on prior knowledge or from candidate marker genes for each cluster suggested by VirtualCytometry. Next, the interactive interface enables users to choose a threshold value with a range slider and see whether a user-selected marker gene can segregate cells that express the marker at a level above the threshold level into a localized region of the two-dimensional latent space. These two subsets divided according to the marker expression level may belong to different states of cellular differentiation or activation.
3. *Identification of DEGs between the cell subsets*: DEGs between the two cell subsets are identified by non-parametric Wilcoxon test. Significant DEGs are sorted by fold-changes in expression. Highly ranked DEGs are likely associated with cellular differentiation or activation. Because researchers are often more interested in TFs and cellular surface receptors that have a higher potential of therapeutic application, we annotated DEGs from a precompiled list of TFs and surface proteins.
4. *Output plots to display the relationship between the marker gene and DEGs in the given cell population*: The correlation between two genes is presented in a dot plot with density contour lines and violin plots.

### 2.6 Hypothesis-test module

This module allows users to test whether two query genes are correlated with each other across cells for different functional states in a given cell population.

1. *Selection of a cell population for analysis*: This step is the same as that used for the ‘Discovery module’.
2. *Selection of two genes for analysis*: If one query gene is a marker known to be involved in the functional transition process within the selected cell population, users can test whether the other query gene is associated with this process based on its expression correlation across cells.
3. *Output plots to display the relationship between two genes in the given cell population*: The relationship between a marker and candidate gene is visualized as a dot plot with density contour lines. If a correlation is observed between the two genes, the candidate gene is highly likely to be associated with the cellular differentiation process involving the marker gene. Violin plots cannot be generated because there are no cellular subsets divided according to the marker gene expression.

## 3 Results

### 3.1 VirtualCytomety identified checkpoint molecules and transcription factors involving human T-cell exhaustion in tumor

Recently, the application of cancer immunotherapy using immune checkpoint inhibitors for reinvigorating tumor-specific T-cell activity and improving clinical outcomes has greatly increased. However, it remains challenging to achieve a long-term benefit for many patients partly because intratumoral T-cell exhaustion can occur (Thommen and Schumacher, 2018). T cells are activated by tumor antigens presented by DCs in the lymph node, and then infiltrate into tumor tissue with the aid of various factors. However, when tumor-infiltrating T cells encounter the immunosuppressive tumor microenvironment, a transcriptional program is initiated that leads to an exhausted state (Chen and Mellman, 2013). Exhausted CD8^+^ T cells are composed of heterogeneous cell populations in various differentiation states (Hashimoto *et al.*, 2018). Upregulation of programmed cell death-1 (PD-1) on T cells is a major phenotype of T-cell exhaustion. Importantly, exhausted CD8^+^ T cells with an intermediate level of PD-1 can be reinvigorated by PD-1 blockade, whereas those with high level PD-1 expression cannot (Blackburn *et al.*, 2008). Therefore, characterizing the phenotypes and cellular differentiation programs of exhausted CD8^+^ T cells may lead to improvements in cancer immunotherapy (Hashimoto *et al.*, 2018).

**Fig. 3.**
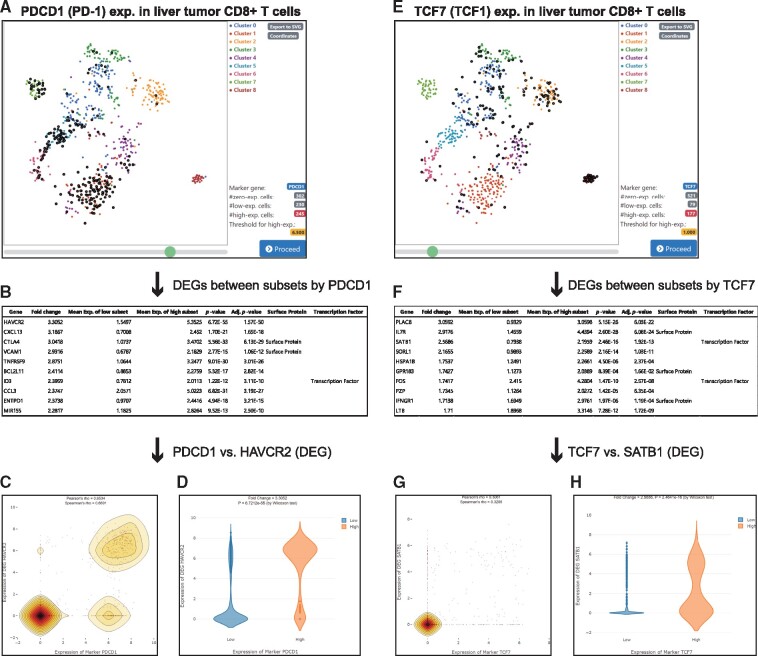
Identification of checkpoint molecules and transcription factors for T-cell exhaustion using VirtualCytometry. (**A**) tSNE plot for cytotoxic T cells from liver tumor. Each dot represents a single cell. Large bold dots represent individual cells expressing a user-selected marker gene, PDCD1, at higher than the threshold level (6.5 in this case study). (**B**) Top 10 DEGs (by Wilcoxon test) with highest fold-changes between PDCD1^low^ and PDCD1^high^ subsets. (**C**) Dot plot with density contour lines showing expression relationship between marker PDCD1 and HAVCR2, which was the top DEG between the PDCD1^low^ and PDCD1^high^ subsets. (**D**) Violin plots of HAVCR2 expression for PDCD1^low^ subset (blue) and PDCD1^high^ subset (red). (**E**) Same as for (A) except using TCF7 as a marker gene (expression threshold of 1.0). (**F**) Top 10 DEGs with highest fold-changes between TCF7^low^ and TCF7^high^ subsets. (**G**) Dot plot with density contour lines showing expression relationship between the marker TCF7 and SATB1, which is the third-ranked DEG. (**H**) Violin plots of SATB1 expression for TCF7^low^ subset (blue) and TCF7^high^ subset (red). (Color version of this figure is available at *Bioinformatics* online.)

We hypothesized that genes associated with CD8^+^ T-cell exhaustion express significantly different levels of genes between early and late stage exhausted cells. To test the hypothesis, we first selected a dataset of cytotoxic T cells from a liver tumor (GSE98638) (Zheng *et al.*, 2017), which were visualized by tSNE. Next, we used the PDCD1 gene which encodes PD-1 as a marker to classify the cells into two subsets for different stages of exhaustion. We found that the optimal threshold of PDCD1 expression was 6.5, at which cells with above threshold level of PDCD1 expression were segregated into a local region of the two-dimensional latent space of the tSNE plot (Fig. 3A). DEGs between the PDCD1^high^ subset and PDCD1^low^ subset were identified using non-parametric Wilcoxon test. We detected many known checkpoint molecules such as HAVCR2 (TIM-3) and CTLA4 among the highly ranked DEGs (Fig. 3B), suggesting that other significant DEGs are associated with CD8^+^ T-cell exhaustion. As expected, data visualization with a dot plot and violin plots supported the correlation between the marker gene (PDCD1) and an identified DEG (HAVCR2) (Fig. 3C and D).

In contrast to PDCD1 (PD-1), TCF7 (TCF1) is a marker of stem cell-like proliferative PD-1^low^ CD8^+^ T cells (Im *et al.*, 2016). Notably, cells with TCF7 expression levels above the threshold (threshold value was 1.0) were segregated into the tSNE space (Fig. 3E) as observed for the PDCD1 marker, but into a different region (compared with Fig. 3A). We identified different DEGs between TCF7^high^ subset and TCF7^low^ subset (threshold value of 1.0) (Fig. 3F). Interestingly, a highly ranked DEG, SATB1, was recently reported as a negative regulator of PDCD1 expression that prevents T-cell exhaustion (Stephen *et al.*, 2017). We found that the expression of CTF7 and SATB1 was correlated based on the dot plot and violin plots (Fig. 3G and H). Collectively, these results demonstrate that VirtualCytometry can identify signaling molecules and transcription factors involved in regulating the transition of immune cells between different functional states (e.g. effector T cells and exhausted T cells) using scRNA-seq data.

### 3.2 VirtualCytometry identified signaling molecules and modulating factors involved in mouse DC activation by pathogens

Although investigation of immune cells from primary normal and disease tissues have become easier by using scRNA-seq technology, single-cell transcriptome profiling for some tissues in human remains limited. For example, obtaining single-cell expression data for lymphocytes under development in the bone marrow or under activation by microbes in the spleen in human is limited by ethical considerations. Indeed, scRNA-seq data for human immune cells are heavily biased towards peripheral blood mononuclear cells. Thus, scRNA-seq data of immune cells for such lymphoid organs were mostly obtained from mouse. We compiled various scRNA-seq data for mouse immune cells derived from the bone marrow and spleen and deposited this information in VirtualCytometry.

**Fig. 4.**
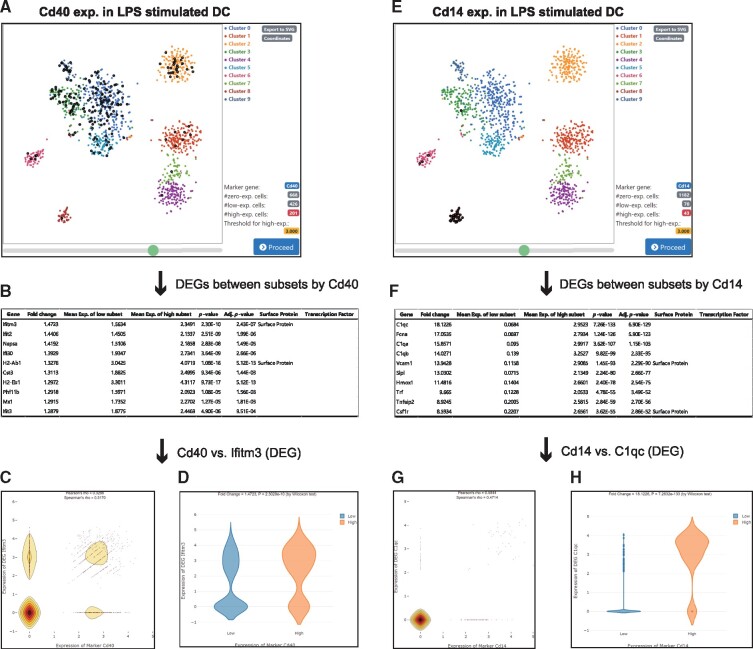
Identification of genes involved in modulating mouse DC maturation and activation using VirtualCytometry. (**A**) tSNE plot for DCs from mouse spleen. Each dot represents a single cell. Large bold dots represent individual cells expressing a user-selected marker gene, Cd40, at higher than the threshold level (3.0 in this case study). (**B**) Top 10 DEGs (by Wilcoxon test) with highest fold-changes between Cd40^low^ and Cd40^high^ subsets. (**C**) Dot plot with density contour lines showing expression relationship between the marker Cd40 and Ifitm3, which was the top-ranked DEG. (**D**) Violin plots of Ifitm3 expression for Cd40^low^ subset (blue) and Cd40^high^ subset (red). (**E**) Same as in (A) except using Cd14 as a marker gene (expression threshold of 3.0). (**F**) Top 10 DEGs with highest fold-changes between Cd14^low^ and Cd14^high^ subsets. (**G**) Dot plot with density contour lines showing expression relationship between the marker Cd14 and C1qc, which was the top DEG. (**H**) Violin plots of C1qc expression for Cd14^low^ subset (blue) and Cd14^high^ subset (red). (Color version of this figure is available at *Bioinformatics* online.)

To demonstrate the usefulness of VirtualCytometry for studying mouse immune cells, we tested its ability to predict genes involved in DC activation and maturation upon stimulation by pathogen-associated molecular patterns such as lipopolysaccharide (LPS). From the precompiled scRNA-seq datasets, we selected GSE54006 which contains gene expression data for 1295 DCs from the mouse spleen stimulated with LPS (Jaitin *et al.*, 2014). As a marker, we used Cd40, which regulates multiple signaling pathways involved in DC activation (Ma and Clark, 2009). We found that Cd40^high^ cells (threshold value of 3.0) were enriched in only some clusters of LPS-stimulated DCs, indicating that not all stimulated DCs had the same activation status (Fig. 4A). We identified DEGs between the Cd40^high^ and Cd40^low^ subsets based on Wilcoxon test (Fig. 4B). VirtualCytometry retrieved well-known interferon-stimulated genes (ISGs) (Fensterl and Sen, 2015; Schneider *et al.*, 2014) such as Ifitm3, Ifit2, Ifi30, Mx1, Ifit3, Ifit1 and Irf7 among the highly ranked DEGs between the Cd40^high^ and Cd40^low^ subsets. We also observed a correlation between the expression of Cd40 and the ISGs with a dot plot and violin plots provided by the web tool (Fig. 4C and D).

Cd14, a marker of monocytes, was lost in activated DCs but enriched in a cluster depleted by Cd40 (Fig. 4E). We found that complement proteins such as C1qa, C1qb and C1qc were upregulated in Cd14^high^ DCs compared to in Cd14^low^ DCs (threshold value of 3.0) (Fig. 4F). DCs containing C1q (C1qDC) were reported to show defects in the production of interferon, suggesting that C1q modulates DCs (Castellano *et al.*, 2007). We found that the expression of Cd14 and complement proteins was correlated based on the dot plot and violin plots (Fig. 4G and H). Collectively, these results suggest the VirtualCytometry can use scRNA-seq data to identify novel genes associated with mouse DC maturation.

## 4 Discussion

VirtualCytometry mimics the method used to analyze data from flow or mass cytometry: dividing cells into subsets using a marker gene for different functional states of differentiation stages and then identifying new genes associated with the transition between different cellular states based on DEGs between subsets. However, VirtualCytometry has some advantages over conventional cytometry analysis. First, it can provide a much more comprehensive picture of the molecular phenotype of cells, as scRNA-seq technology can theoretically measure the expression of the whole genome. In contrast, the number of cellular features measured by conventional cytometry currently does not exceed 50 because of the limited resolution and antibody availability (Spitzer and Nolan, 2016). Second, an unbiased genome-wide survey of molecular features can provide opportunities for unexpected discoveries. Studies using conventional cytometry start with a set of features that are typically selected based on a prior hypothesis. Exhaustively searching of the entire feature space may reveal unpredicted information. Third, numerous scRNA-seq datasets are present in public repositories that will exponentially grow in the future. This data availability, which does not exist for cytometry studies, will facilitate novel discoveries through data reuse.

VirtualCytometry also has some intrinsic and technical limitations compared to conventional cytometry. First, it cannot account for post-transcriptional regulations, as it detects functional features at the transcript level rather than at the protein level. Second, the detection sensitivity is low, commonly resulting in dropouts for ∼90% of readouts in each experiment. This can be resolved by improving the molecule capture efficiency during scRNA-seq sample preparation.

Currently, VirtualCytometry contains precompiled scRNA-seq data for 111 and 115 cell populations from human and mouse, respectively, in various tissues and disease conditions. Because the number of public scRNA-seq datasets grows with a rapidly increasing rate, a significant effort should be placed on the continuous update of new datasets. In addition, although only JingleBells and GEO were considered as sources of public data for this version of release, we need to expand our collection of scRNA-seq datasets to other depositories such as European Nucleotide Archive (ENA) (Harrison *et al.*, 2019) and DNA Data Bank of Japan (DDBJ) (Mashima *et al.*, 2017) in the future.

We successfully identified signaling molecules and transcription factors involved in the transition between the different functional states of T cells and DCs in tumor and lymphatic organs; thus, candidate genes generated by VirtualCytometry are likely associated with the query cellular transition processes. Although VirtualCytometry allows maker selection based on DEGs for cellular subpopulations based on Louvain clustering, it still uses supervised approaches to predictions currently. Therefore, developers and users need to be aware of benefit by having some degree of domain knowledge.

In the future, VirtualCytometry can be extended to study cell differentiation in other body systems by including scRNA-seq from relevant tissue contexts (e.g. study of the differentiation of neurons derived from brain tissues). In addition, enabling comparative analysis between two distinct but related datasets (e.g. comparison between wild-type and knockout cells derived from the same study or between the same type of cells derived from different studies) would enhance the utility of this tool. In conclusion, VirtualCytometry will greatly enhance the utilization of public scRNA-seq data and facilitate studies of immune cell differentiation and eventually the development of novel therapeutics for immune-related diseases including cancer.

## Funding

This work was supported by the National Research Foundation of Korea (NRF) grant funded by the Korean Government (MSIT) [NRF-2018M3C9A5064709, NRF-2018R1A5A2025079 and NRF-2019M3A9B6065192].

*Conflict of Interest*: none declared.
